# The Effects of Brain Magnetic Resonance Imaging Indices in the Association of Olfactory Identification and Cognition in Chinese Older Adults

**DOI:** 10.3389/fnagi.2022.873032

**Published:** 2022-07-05

**Authors:** Ziyi Tan, Yingzhe Wang, Heyang Lu, Weizhong Tian, Kelin Xu, Min Fan, Xiaolan Zhao, Li Jin, Mei Cui, Yanfeng Jiang, Xingdong Chen

**Affiliations:** ^1^State Key Laboratory of Genetic Engineering, Zhangjiang Fudan International Innovation Center, School of Life Sciences, Human Phenome Institute, Fudan University, Shanghai, China; ^2^Fudan University Taizhou Institute of Health Sciences, Taizhou, China; ^3^Department of Neurology, Huashan Hospital, Fudan University, Shanghai, China; ^4^Department of Medical Imaging, Taizhou People’s Hospital Affiliated to Nantong University, Taizhou, China; ^5^The Key Laboratory of Public Health Safety of Ministry of Education, Department of Biostatistics, School of Public Health, Fudan University, Shanghai, China; ^6^Taixing Disease Control and Prevention Center, Taizhou, China; ^7^Taizhou Disease Control and Prevention Center, Taizhou, China; ^8^International Human Phenome Institute (Shanghai), Shanghai, China; ^9^Yiwu Research Institute of Fudan University, Yiwu, China

**Keywords:** olfactory identification, cognitive function, dementia, mild cognitive impairment, brain atrophy

## Abstract

**Background:**

Olfactory identification dysfunction frequently occurs in individuals with cognitive decline; however, a pathological mechanism linking the two has not been discovered. We aimed to study the association between olfactory identification and cognitive function, and determine the effects of brain regions atrophy therein.

**Methods:**

A total of 645 individuals (57.5% were female) from the Taizhou Imaging Study, who underwent cognitive and olfactory identification measurements, were included. A subsample of participants underwent brain magnetic resonance imaging (*n* = 622). Cognition was assessed with a neuropsychological battery. Olfactory identification was measured using a 12-item Sniffin’ Sticks test. Beta and logistic regressions were used to elucidate the association between olfactory identification and cognition, and the effects of brain regions atrophy in this association.

**Results:**

Dementia was diagnosed in 41 (6.4%) individuals (mean age = 64.8 years), and mild cognitive impairment (MCI) in 157 (24.3%) individuals (mean age = 64.4 years). Olfactory identification was associated with MMSE and MoCA (both *P* < 0.001) and specific cognitive domains (memory, executive function, visuospatial function, and language; all *P* < 0.05). Higher olfactory identification was associated with lower likelihood of MCI and dementia (*P* < 0.05). The amygdala volume was significantly related to olfactory identification, MMSE, MoCA, and language, and could attenuate the association between olfactory identification and cognitive function.

**Conclusion:**

The association between olfactory identification and cognition can be partly attributable to differences in amygdala volume, suggesting that the amygdala could be a shared neural substrate that links olfactory identification and cognitive function. Limitations of this study include that all these results were based on a cross-sectional study.

## Introduction

Dementia is being a great challenge for health and social care in China. It occurs mainly in residents older than 65 years and the prevalence increases with age (Jia et al., 2020). Nowadays, disease-modifying treatment is still not available, however, it is possible to predict the progression of dementia with biomarkers (Livingston et al., 2017). Olfactory identification is associated with cognitive function, including global and domain-specific cognitive function (Liang et al., 2016; Vassilaki et al., 2017; Dahmani et al., 2018; Palta et al., 2018; Yahiaoui-Doktor et al., 2019). Olfactory identification dysfunction is frequently observed in populations with cognitive decline and cognitive dysfunction (Devanand, 2016; Fullard et al., 2016; Roberts et al., 2016; Roalf et al., 2017; Walker et al., 2021). For example, it was reported previously that olfactory identification dysfunction appears in early Alzheimer’s disease (AD) stages and shows accelerated progression over the course of dementia (Serby et al., 1991; Djordjevic et al., 2008). Thus, olfactory identification is considered a non-invasive marker for identifying preclinical stages of mild cognitive impairment (MCI) or dementia and predicting disease progression (Devanand et al., 2015; Vassilaki et al., 2017). However, the mechanisms that explain the association between olfactory identification and cognitive dysfunction remain unclear and the pathway underlying requires more investigation.

Disruption of brain structure is associated with cognitive decline and dementia in elderly adults (Apostolova et al., 2010; Wang et al., 2018). Atrophy of brain regions like hippocampus and amygdala is associated with diminished performance in olfactory function test both in healthy individuals and those with dementia (Smitka et al., 2012; Lian et al., 2019). Brain atrophy occurred in people with olfactory dysfunction and people with cognitive dysfunction (Dintica et al., 2019). In dementia population, the hippocampus and amygdala are smaller in those with olfactory dysfunction than those with normal olfactory function, suggesting that hippocampus and amygdala atrophy may be shared by olfactory function and cognitive function changes (Lian et al., 2019). It seems that brain atrophy both correlates with these two clinical manifestations. Therefore, exploring the effects of brain structure in the association of olfactory identification and cognition may help to understand the underlying mechanisms. However, till now, how the structural damage of brain regions relate to cognitive dysfunction in olfactory dysfunctions has remained to be studied. Thus, we hypothesized that impaired olfactory identification is associated with cognitive dysfunction, and such association might be partly attributable to brain atrophy. In this study, we aimed to replicate previous findings on the associations between olfactory identification and cognitive function, including scores of global and domain-specific cognitive function, as well as cognitive dysfunction (MCI and dementia); elucidate the associations between olfactory identification, cognitive function, and brain magnetic resonance imaging (MRI) indices; and determine whether differences in brain MRI indices might explain the association between olfactory identification and cognitive function, by using data from a community -based study of rural Chinese older adults.

## Materials and Methods

### Study Design and Participants

Participants in this study were chosen from the Taizhou Imaging Study (TIS), an ongoing well-characterized community-based neuroimaging cohort nested in the Taizhou Longitudinal Study (TZL) that aims to monitor the risk factors and progression of dementia and cerebrovascular disease in a rural Chinese population. The study design of TIS has been described previously (Jiang et al., 2021). Briefly, 904 individuals aged 55–65 years without a history of physician-diagnosed stroke, dementia, cancer, and other severe diseases were enrolled and received baseline examinations including epidemiological questionnaire survey, physical and clinical examination (e.g., multimodal brain MRI), and neuropsychological and olfactory identification assessment from 2013 to 2018. In annual cognitive follow-ups, the TIS conducted more comprehensive cognitive function assessments, and olfactory identification tests (Jiang et al., 2021). In the present study, we performed a cross-sectional analysis using data from the first round of follow-ups of the TIS. As shown in Supplementary Figure 1, 904 individuals were included in TIS at baseline. After the exclusion of individuals without taking olfaction or cognitive function test due to various reasons, including death, refusal, not able to cooperate, etc. (*N* = 219) and data missing (*N* = 2), 683 individuals left. Among them, two individuals had history of nasal surgery, 12 individuals had nasal disease, one individual got cold and 22 individuals had unqualified data due to poor cooperation, leaving 645 individuals (622 individuals had MRI data) with full of cognition assessment and involved in final analysis. The TIS was approved by the Ethics Committee of the School of Life Sciences, Fudan University, and Fudan University Taizhou Institute of Health Sciences (institutional review board approval number: 496 and B017, respectively). Written informed consent was obtained from each participant before data and biospecimen collection.

### Neuropsychological Measurements and Diagnosis of Mild Cognitive Impairment and Dementia

A neuropsychological test battery was used to assess participants’ cognitive function, as described previously (Jiang et al., 2021). Briefly, several neuropsychological tests generated from developed countries, which covered global cognition, memory, executive function and attention, visuospatial function, and language, were adapted, normalized, and validated to suit Chinese culture (Jiang et al., 2021). The neuropsychological tests used in this study have been validated in Chinese population and normative data has been published previously (Ding et al., 2015). The test battery included (1) Mini-Mental State Examination (MMSE); (2) Beijing version Montreal Cognitive Assessment (MoCA); (3) Chinese version of Auditory Verbal Learning Test (AVLT); (4) Modified Fuld Object Memory Evaluation (FOME); (5) Clock Drawing Test (CDT); (6) Conflicting Instructions Task (Go/No Go task); (7) Trail-Making Test A & B; and (8) Animal Fluency Test (AFT). According to the education levels of the participants, two versions of tests were assessed separately: (1) to (3) and (5)–(8) were used for participants with ≥6 years of formal education (participants who completed primary education), while (1), (2), and (4)–(8) were used for participants with <6 years of education (Cui et al., 2020; Jiang et al., 2021). Among them, The Auditory Verbal Learning Test was adapted from California Verbal Learning Test and The Modified Fuld Object Memory Evaluation was adapted from the Fuld Object Memory Evaluation (Ding et al., 2015). All the assessments were conducted by neurologists, neuropsychologists, and trained technicians with over 3 years’ experience independently. Dementia and MCI were diagnosed by a committee consisting of expert neurologists and neuropsychologists, according to the Diagnostic and Statistical Manual of Mental Disorders, 4th edition (DSM-IV) (Blashfield et al., 2014) and Petersen RC’s criteria (Petersen, 2004), respectively, depending on each participant’s cognitive measurements, clinical manifestations, and specific neuroimaging features (Ding et al., 2015; Cui et al., 2020; Jiang et al., 2021).

### Measurement of Olfactory Identification

Olfactory identification was measured using the 12-item Sniffin’ Sticks screening test (Palta et al., 2018; Jiang et al., 2021). Each participant was asked to smell 12 common odorants (orange, leather, cinnamon, peppermint, banana, lemon, licorice, coffee, cloves, pineapple, rose, and fish) one at a time, in different orders, for longer than 3–4 s, and then asked to identify the odor from four answer choices. Three rounds of separate tests were performed for the left nostril, right nostril, and nostrils on both sides. When the nostril on one side was under test, participants were asked to block the other nostril. To accommodate the differences in education levels of the participants, the list of odors was represented as pictures. The administrators of the test were blind for the cognitive status of each participant. Before the tests, the participants were asked about olfactory history, previous diseases, and occupations that could have an influence on olfactory identification. People who have these problems were excluded. One point was given for each correctly identified odor, and the possible score ranged from 0 to 12. Score on both sides was calculated for olfactory identification. A higher test score indicated better olfactory identification.

### Magnetic Resonance Imaging Acquisition and Measurements

All the participants of the TIS were scanned on a 3.0-T multi-modality brain MRI scanner (Magnetom Verio Tim scanner; Siemens, Erlangen, Germany) at baseline. The protocol and MRI sequence parameters have been reported previously (Wang et al., 2019; Cui et al., 2020; Jiang et al., 2021). FreeSurfer software (v6.0.0) was used to estimate the volume of brain regions on T1-weighted images (Desikan et al., 2006). We measured the volumes of the hippocampus, thalamus, amygdala, cerebral cortex, and other subcortical structures correlated to cognitive function. Total intracranial volume (TIV) and volumes of cerebrospinal fluid (CSF), ventricle, white matter, and gray matter were used to determine global atrophy. All the brain region volumes were standardized by z-score. Further, we obtained and log-transformed volume of white matter hyperintensity (WMH) by using SPM8 (Schmidt et al., 2012).

### Covariates

Demographics (age, sex, and years of education) and lifestyle characteristics (smoking and alcohol consumption) data were collected using a detailed epidemiological questionnaire. Medical history of hypertension, diabetes, and hyperlipidemia were obtained from diagnoses by physicians, and physical examination (Jiang et al., 2021).

### Statistical Analyses

#### Statistical Analyses for Association of Olfactory Identification and Cognition

Continuous variables were presented as mean (standard deviation, *SD*) or median (interquartile range, IQR) and categorical variables were presented as frequencies (%). The normality of continuous variables was evaluated using Shapiro-Wilk test. Kruskal-Wallis test and Pearson chi-square test were used to compare differences among groups of different cognitive status when appropriate. For bounded continuous variables with skewed distribution (MMSE, MoCA, AVLT, FOME, and Conflicting Instructions Task), we transformed the scores into beta distribution (0–1) and then used beta regression to assess the association between olfactory identification with global cognition, memory and executive function (Zou et al., 2010; Martinelli-Boneschi et al., 2013; Jiang et al., 2019). For ordinal categorical variable (CDT), we conducted ordinal logistic regression to evaluate the associations of olfactory identification and visuospatial function (Paula et al., 2013). For continuous variable (AFT), generalized liner regression was used (Caldwell et al., 2019). The regression analyses were conducted in all participants. We used multinomial logistic regression to estimate the association of olfactory identification score and cognitive dysfunction, using cognitively normal as a reference.

#### Statistical Analyses for Association Between Olfactory Identification, Cognitive Function, and Brain Volume

For the reasons mentioned above, the associations between brain region volumes and olfactory identification, global cognition, and domain-specific cognitive function were assessed using beta regression, ordinal logistic regression and generalized liner regression, respectively. Since we did the analyses between 14 brain regions with olfactory identification and cognitive function, to account for multiple comparison correction to reduce false positivity, we used a false discovery rate (FDR) correction and interpreted FDR–corrected *P*-values < 0.05 as significance levels for the associations among olfactory identification, cognition, and brain region volumes. Finally, to determine whether differences in brain MRI indices might explain the association between olfactory identification and cognition in all participants, brain region volumes associated with both olfactory identification and cognitive function were included in beta regression or generalized liner regression models to assess whether they could attenuate the association between olfactory identification and cognition (Rosso et al., 2017). The attenuation of the association was evaluated using R package (mediation) with 10,000 repetitions. We also stratify the association between olfactory identification and cognition according to the median of amygdala volume. All regression analyses were fitted in two models: Model 1 was adjusted for age, sex, and years of education; Model 2 was further adjusted for hypertension, diabetes, hyperlipidemia, smoking, and alcohol consumption (time interval and standardized TIV was added in both models when brain region volumes were included). Age, sex, and education were reported to be associated with cognitive function previously. Hypertension, diabetes and hyperlipidemia were also reported as risk factors for cognitive dysfunction (Livingston et al., 2017; Barthold et al., 2020). Time interval between brain MRI and cognitive assessment and standardized TIV were also adjusted in the model to eliminate the effects due to individual differences. Differences were considered statistically significant at *P* < 0.05. All statistical analyses were performed using R program (R core team, Version 3.6.1).

## Results

### Characteristics of the Study Population

The demographic and olfactory and cognitive performance characteristics of the study population (*n* = 645) are presented according to cognitive status in Table 1. Among the 645 followed-up TIS participants, dementia was diagnosed in 41 (6.4%) individuals, and MCI in 157 (24.3%) individuals at the first round of cognitive follow-up (Jiang et al., 2021). The individuals with dementia and MCI tended to be female and older. Participants with cognitive dysfunction were likely to have obtained less education years and have higher rate of hypertension. As expected, participants with MCI and dementia had significant lower neuropsychological scores, including global cognitive function (MMSE and MoCA) and domain-specific cognitive function (all *P* < 0.001). Moreover, in olfactory identification tests, cognitively normal individuals (median: 6, IQR: 5, 8) had a higher median score and showed better performance than individuals with dementia (median: 5, IQR: 3, 6).

**TABLE 1:** Characteristics of the study participants.

Characteristics	Cognitively normal *N* = 447	MCI *N* = 157	Dementia *N* = 41	*P*-value
**Demographics**				
Age, mean (*SD*), years	63.7 (3.2)	64.4 (3.0)	64.8 (3.1)	0.033
Female	237 (53.0)	100 (63.7)	34 (82.9)	< 0.001
Years of education	6 (2, 9)	2 (0, 8)	0 (0, 3)	< 0.001
**Pre-existing comorbidity and lifestyle**				
Hypertension	231 (51.7)	101 (64.3)	23 (56.1)	0.421
Diabetes	63 (14.1)	15 (9.6)	5 (12.2)	0.024
Hyperlipidemia	250 (55.9)	79 (50.3)	24 (58.5)	0.037
Smoker	136 (30.4)	38 (24.2)	5 (12.2)	0.341
Alcohol drinker	149 (33.3)	41 (26.1)	7 (17.1)	0.023
**Olfactory function**				
Olfactory identification	6 (5, 8)	6 (4, 7)	5 (3, 6)	< 0.001
**Cognitive function**				
MMSE	26 (23, 28)	21 (16, 25)	13 (9, 15)	< 0.001
MoCA	18 (14, 22)	12 (9, 17)	7 (4, 8)	< 0.001
Short delay recall	8 (6, 10)	7 (4, 8)	7 (5, 8)	< 0.001
Long delay recall	8 (6, 10)	7 (4, 8)	6 (5, 8)	< 0.001
Executive function	2.5 (2, 3)	2 (1.5, 2.5)	1 (1, 1.5)	< 0.001
Visuospatial function	2 (1, 3)	1 (0, 2)	0 (0, 1)	< 0.001
Language	12 (10.50, 15)	10 (8, 12)	7 (4, 10)	< 0.001

*Values are median (interquartile range) or N (%), except for indicated.*

*SD, Standard deviation; MMSE, Mini-Mental State Examination; MoCA, Montreal Cognitive Assessment; MCI, Mild cognitive impairment.*

### Association of Olfactory Identification and Cognitive Function

We first assessed the association between olfactory identification and cognitive function (Table 2). Olfactory identification score was associated with MMSE and MoCA (Beta = 0.075 and 0.107, respectively; both *P* < 0.001). Results were robust to further adjustment for smoking, alcohol consumption, and comorbidities (Model 2, both *P* < 0.001). In the fully adjusted Model 2, olfactory identification score was also associated with specific cognitive domain (short delay recall: Beta = 0.046, *P* = 0.018; long delay recall: Beta = 0.065, *P* = 0.001; executive function: Beta = 0.066, *P* = 0.011; visuospatial function: Beta = 0.159, *P* < 0.001; language: Beta = 0.032, *P* < 0.001).

**TABLE 2:** Association of olfactory identification with cognitive function.

Cognitive function	Model 1	*P*-value	Model 2	*P*-value
MMSE	0.075 (0.016)	< 0.001	0.073 (0.016)	< 0.001
MoCA	0.107 (0.013)	< 0.001	0.106 (0.013)	< 0.001
Short delay recall	0.045 (0.020)	0.022	0.046 (0.019)	0.018
Long delay recall	0.066 (0.020)	0.001	0.065 (0.020)	0.001
Executive function	0.068 (0.026)	0.008	0.066 (0.026)	0.011
Visuospatial function	0.160 (0.038)	< 0.001	0.159 (0.038)	< 0.001
Language	0.033 (0.006)	< 0.001	0.032 (0.006)	< 0.001

*Values are estimated coefficients (standard error). Model 1 was adjusted for age, sex, and years of education; Model 2 was further adjusted for hypertension, diabetes, hyperlipidemia, smoking and alcohol consumption.*

*MMSE, Mini-Mental State Examination; MoCA, Montreal Cognitive Assessment.*

### Association Between Olfactory Identification, Cognitive Function, and Brain Volume

**FIGURE 1:**
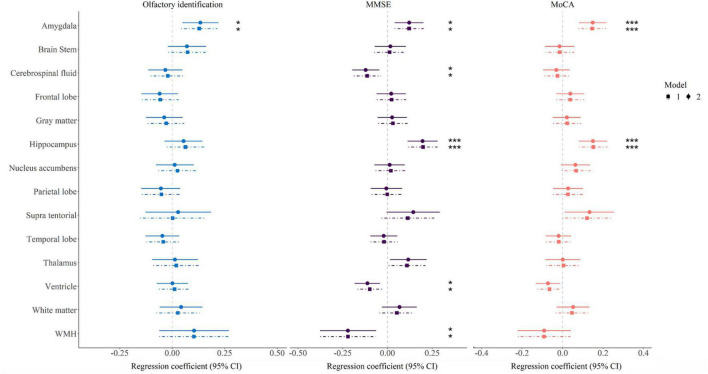
Forest map showing association between brain region volume and olfactory identification and cognitive function. Model 1 was adjusted for age, sex, years of education, time interval, and standardized total intracranial volume; Model 2 was further adjusted for hypertension, diabetes, hyperlipidemia, smoking and alcohol consumption. The significance threshold was set at *P < 0.05 and ***P < 0.001 after correcting for the false discovery rate. MMSE, Mini-Mental State Examination; MoCA, Montreal Cognitive Assessment; WMH, white matter hyperintensity.

As is shown in Figure 1 and Supplementary Table 2, olfactory identification score was associated with the volume of amygdala (of the 14 brain structures tested), in Model 1. Hypertension, diabetes, hyperlipidemia, smoking, and alcohol consumption, did not modify the association between olfactory identification and the volume of amygdala (Beta = 0.134, FDR = 0.028). After full adjustment (Model 2), MMSE results were significantly associated with the volumes of the amygdala (Beta = 0.121, FDR = 0.019), CSF (Beta = -0.122, FDR = 0.013), hippocampus (Beta = 0.196, FDR < 0.001), ventricle (Beta = -0.112, FDR = 0.013), and WMH (Beta = -0.221, FDR = 0.023), as shown in the middle column in Figure 1 and Supplementary Table 2. In addition, MoCA was associated with the volumes of the amygdala (Beta = 0.149, FDR < 0.001), hippocampus (Beta = 0.150, FDR < 0.001) in Model 2 (right column in Figure 1 and Supplementary Table 2). For domain-specific cognitive function, the ventricle and WMH volumes were related to long delay recall, and hippocampal volume was correlated with executive function with nominal *P* < 0.05 in Model 2, however, only the amygdala volume (Beta = 0.046, FDR = 0.028) was significantly associated with language after multiple comparison correction (Supplementary Table 3).

### Association of Olfactory Identification With Cognitive Function Before and After Adjustment for Brain Magnetic Resonance Imaging Markers

As shown in Table 3, olfactory identification was associated with MMSE (Beta = 0.068, *P* < 0.001), MoCA (Beta = 0.098, *P* < 0.001), and language (Beta = 0.332, *P* < 0.001). When amygdala volume was introduced into the model, the associations were attenuated but remained significant between olfactory identification and MMSE (Beta = 0.066, *P* < 0.001, 9.5% attenuation), MoCA (Beta = 0.095, *P* < 0.001, 5.7% attenuation), and language (Beta = 0.322, *P* < 0.001, 5.9% attenuation), which indicated that these associations were attributable, at least in part, to differences in amygdala volume.

**TABLE 3:** Association of olfactory identification with cognitive function before and after adjustment for amygdala.

	MMSE			MoCA			Language		
	Beta (SE)	*P*-value	Attenuation	Beta (SE)	*P*-value	Attenuation	Beta (SE)	*P*-value	Attenuation
Olfactory identification	0.068 (0.017)	<0.001		0.098 (0.014)	<0.001		0.332 (0.070)	<0.001	
Olfactory identification, adjusted for amygdala	0.066 (0.017)	<0.001	9.5%	0.095 (0.014)	<0.001	5.7%	0.322 (0.070)	<0.001	5.9%

*Values are estimated coefficients (standard error). All models were adjusted for age, sex, years of education, hypertension, diabetes, hyperlipidemia, smoking and alcohol consumption (time interval and standardized total intracranial volume and was added when amygdala volume was included).*

*MMSE, Mini-Mental State Examination; MoCA, Montreal Cognitive Assessment.*

## Discussion

The main findings from this community-based study in rural older Chinese adults can be summarized as follows: (1) a higher olfactory identification score was associated with better global and domain-specific cognitive function; (2) brain atrophy (especially of the amygdala) impaired olfactory identification and global cognition; and (3) the association between olfactory identification and cognition can be partly attributable to differences in amygdala volume. These findings suggest that poor olfactory identification could indicate cognitive dysfunction associated with brain neurodegeneration. Moreover, the olfactory identification score was negatively associated with the presence of MCI [odds ratio (OR): 0.89, 95% confidence interval (CI): 0.81, 0.97] and dementia (OR: 0.79, 95% CI: 0.66, 0.93) in Model 2 (Supplementary Table 1). After stratification according to the median of amygdala volume, in participants with amygdala volume both in the top 50% and the last 50%, the associations between olfactory identification with MMSE, MoCA and language were found significant (Supplementary Table 5). There is an increase in studies focusing on the association between olfaction dysfunctions and cognitive decline in older people. Previous studies have indicated that olfactory identification is associated with global cognitive function and specific cognitive domains, including memory, language, and executive function (Palta et al., 2018; Yahiaoui-Doktor et al., 2019), which was corroborated by our results. Additionally, poor olfactory identification performance was associated with higher risks of MCI and dementia in older people, as reported previously (Liang et al., 2016; Roberts et al., 2016).

Global cognition and specific cognitive domains were associated with several structural brain features/markers, such as volume of the hippocampus, amygdala, thalamus, caudate, gray matter, white matter, and WMH (Apostolova et al., 2010; Wang et al., 2018; Novellino et al., 2019; Oschwald et al., 2019). We also found that hippocampal and amygdala volumes were associated with MMSE and MoCA. The association between the hippocampal volume and cognitive function has been well established. The hippocampus likely plays a crucial part in the cognitive neural circuit, including learning, decision making, declarative, episodic memory (Wikenheiser and Schoenbaum, 2016; Yang and Wang, 2017), and particularly memory formation (Bellmund et al., 2018). Similarly, amygdala atrophy was closely associated with MMSE, and the symptoms increased in severity when the amygdala volume declined in individuals with early AD (Poulin et al., 2011).

Olfactory dysfunctions have recently been linked with brain lesions such as atrophy (Lee et al., 2014; Takeda et al., 2014). Olfactory dysfunction was associated with smaller volumes of the hippocampus, amygdala, entorhinal cortex, fusiform gyrus, temporal pole, and inferior temporal cortex (Dintica et al., 2019; Lian et al., 2019; Wu et al., 2019). The amygdala is involved in the primary and secondary olfactory cortex, which links several structural pathways that modulate olfactory dysfunction (Han et al., 2019). In this study, we found that amygdala atrophy is associated with declining olfactory identification.

Several pathophysiological pathways could affect the association between olfactory identification dysfunction and cognitive decline in aging. In this study, we aimed to elucidate the effect of specific brain MRI indices on the correlation between olfactory identification and cognitive function. Our results suggest that the amygdala volume is associated with cognitive function, and could attenuate the association between olfactory identification and cognitive function, indicating that the amygdala could be a neural substrate that links olfactory identification to cognitive function. In patients with AD, Baek et al. (2020) found that atrophy in brain regions related to olfactory process may have a direct effect on olfactory identification. As an important component of the cognitive and olfactory neural circuits (Devanand, 2016; Herrington et al., 2017), the volume of the amygdala decreased along with cognitive decline (Poulin et al., 2011). The amygdala is associated with emotion and motivation (Janak and Tye, 2015), and in the progression of learning and consolidation with memory structures such as the hippocampus (Herrington et al., 2017). On the other hand, the amygdala also receives neuronal projections directly from the olfactory bulb (Devanand, 2016), making it a crucial part of the olfactory neural circuit. There has been little research on the effects of brain structure in associations between olfactory identification and cognitive function. Our results suggest that the association between olfactory identification and cognitive function is at least partly attributable to amygdala volume, providing a basis for the elucidation of a possible underlying neurological mechanism.

We studied the relationship between olfactory identification and cognitive function by linking olfactory identification to MRI data, and investigated the probable underlying mechanism. The major strength of our study includes the unique sample of rural Chinese older adults as well as the use of the 3.0-T multi-modality brain MRI scanner. However, this study has several limitations. All these results were based on a cross-sectional study so the order in which cognitive dysfunction and olfactory dysfunction occurred cannot be determined, and more community-based longitudinal data are warranted. Continuous follow-ups will be performed as part of the TIS, in which the associations and effects observed could be further evaluated in the future. Further, we only used odor identification to assess olfactory function in this study. However, due to its sensitivity and specificity, the olfactory identification test has been widely used in olfactory function evaluation and cognition-related research (Boesveldt et al., 2008). Finally, our study indicated that the amygdala could be one of the neural links underlying the association between olfaction and cognition, which is a preliminary result for mechanistic studies. Pathological analysis or longitudinal changes in brain properties and network analysis could be measured in future research to assess the level or percentage of brain atrophy and its contribution to get more evidence for mechanisms that explain this association.

## Conclusion

The results of our community-based study indicate that higher olfactory identification is associated with better global cognition and specific cognitive domains, and lower olfactory identification is positively associated with occurrence of cognitive dysfunction, including MCI and dementia. The association between olfactory identification and cognitive function is attenuated by the volume of amygdala, indicating that the amygdala could be one of the neural links underlying this association. These findings should be further validated in larger populations and in longitudinal studies, and could form the basis for the development of a non-invasive and cost-effective screening tool to identify cognitive dysfunction and monitor the progression of dementia.

## Data Availability Statement

The raw data supporting the conclusions of this article will be made available by the authors, without undue reservation.

## Ethics Statement

The studies involving human participants were reviewed and approved by the Ethics Committee of the School of Life Sciences, Fudan University, and Fudan University Taizhou Institute of Health Sciences (institutional review board approval number: 496 and B017, respectively). The patients/participants provided their written informed consent to participate in this study.

## Author Contributions

YJ and XC: concept and design. YW, WT, MF, and XZ: data collection. ZT and YJ: data analyses and manuscript writing. All authors: manuscript proofing and results interpretations.

## Conflict of Interest

The authors declare that the research was conducted in the absence of any commercial or financial relationships that could be construed as a potential conflict of interest. The reviewer XL declared a shared affiliation with the authors ZT, YW, HL, KX, LJ, MC, YJe and XC to the handling editor at the time of review.

## Publisher’s Note

All claims expressed in this article are solely those of the authors and do not necessarily represent those of their affiliated organizations, or those of the publisher, the editors and the reviewers. Any product that may be evaluated in this article, or claim that may be made by its manufacturer, is not guaranteed or endorsed by the publisher.
