# Supplementary figures and images for: The Effects of Brain Magnetic Resonance Imaging Indices in the Association of Olfactory Identification and Cognition in Chinese Older Adults
Source: Front Aging Neurosci. 2022 Jul 5;14:873032. doi: 10.3389/fnagi.2022.873032 (PMC9294318; doi:10.3389/fnagi.2022.873032)

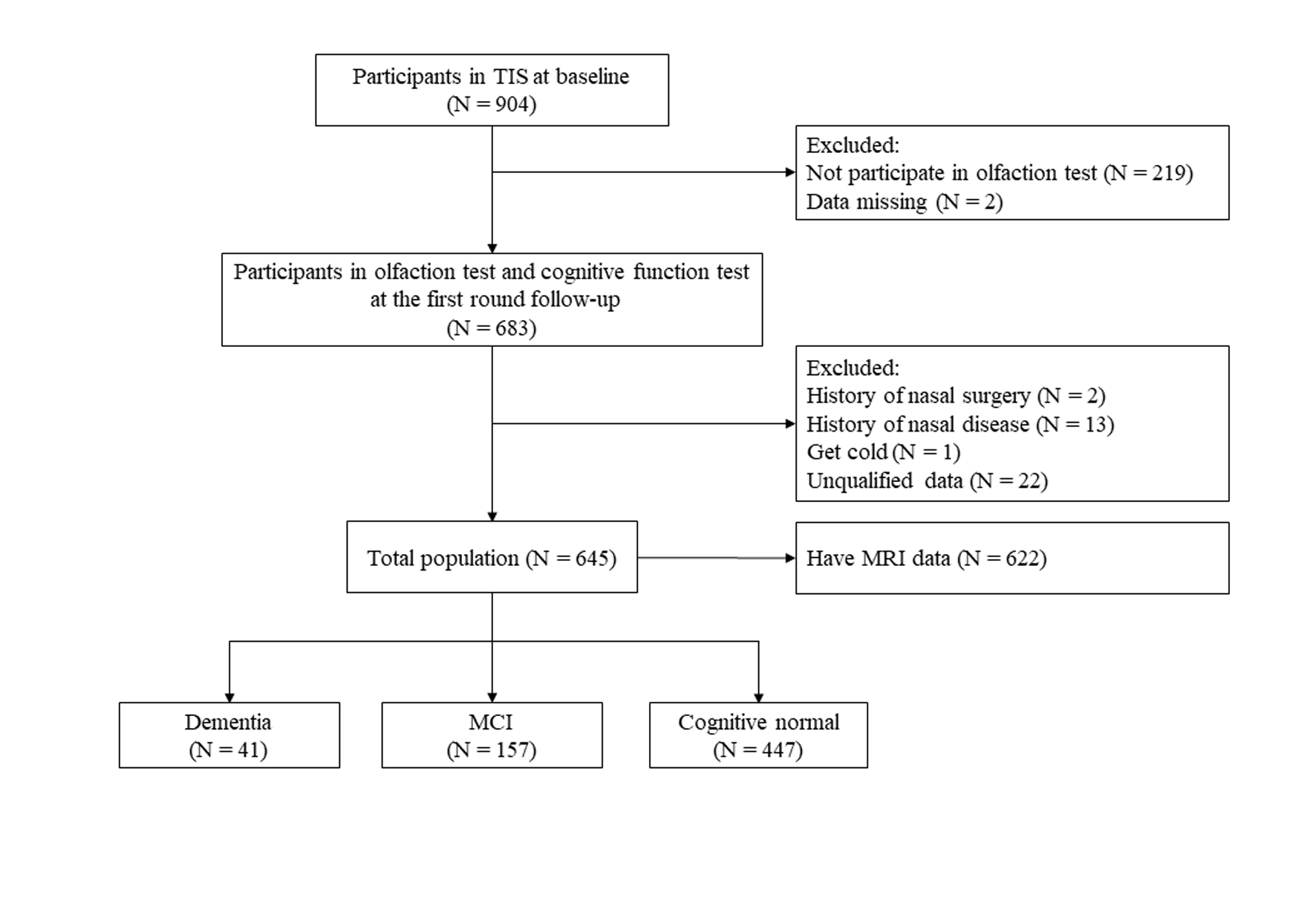

Supplement: Supplementary file 1 [file Image_1.TIF]
