# Supplementary material for: The Effects of Brain Magnetic Resonance Imaging Indices in the Association of Olfactory Identification and Cognition in Chinese Older Adults
Source: Front Aging Neurosci. 2022 Jul 5;14:873032. doi: 10.3389/fnagi.2022.873032 (PMC9294318; doi:10.3389/fnagi.2022.873032)
Supplement: Supplementary file 2 [file Table_1.DOCX]

**Table S1. Association of olfactory identification with cognitive impairment**

| Cognitive Status | Model 1 | *P* value | Model 2 | *P* value |
| --- | --- | --- | --- | --- |
| Cognitively normal | ref |  | ref |  |
| Mild cognitive impairment | 0.89 (0.81, 0.97) | 0.012 | 0.89 (0.81, 0.97) | 0.013 |
| Dementia | 0.79 (0.66, 0.93) | 0.006 | 0.79 (0.66, 0.93) | 0.005 |

Data are presented as odds ratio (95% confidence interval). Model 1 was adjusted for age, sex, and years of education; Model 2 was further adjusted for hypertension, diabetes, hyperlipidemia, smoking and alcohol consumption.

**Table S2 Association of brain region volume with olfactory identification and cognitive function**

| Brain region | Model | Olfactory identification | | |  | MMSE | | |  | MoCA | | |
| --- | --- | --- | --- | --- | --- | --- | --- | --- | --- | --- | --- | --- |
|  |  | Beta (SE) | *P* value | *P* value (FDR) |  | Beta (SE) | *P* value | *P* value (FDR) |  | Beta (SE) | *P* value | *P* value (FDR) |
| Amygdala | 1 | 0.129 (0.044) | 0.003 | 0.028 |  | 0.121 (0.041) | 0.003 | 0.019 |  | 0.147 (0.034) | <0.001 | <0.001 |
|  | 2 | 0.134 (0.044) | 0.002 | 0.028 |  | 0.121 (0.041) | 0.004 | 0.019 |  | 0.149 (0.034) | <0.001 | <0.001 |
| Brain Stem | 1 | 0.073 (0.046) | 0.115 | 0.464 |  | 0.012 (0.044) | 0.780 | 0.823 |  | -0.014 (0.037) | 0.710 | 0.794 |
|  | 2 | 0.070 (0.046) | 0.133 | 0.432 |  | 0.016 (0.044) | 0.716 | 0.800 |  | -0.015 (0.037) | 0.681 | 0.761 |
| Cerebrospinal fluid | 1 | -0.021 (0.041) | 0.605 | 0.732 |  | -0.113 (0.038) | 0.003 | 0.019 |  | -0.025 (0.033) | 0.443 | 0.652 |
|  | 2 | -0.033 (0.041) | 0.428 | 0.581 |  | -0.122 (0.039) | 0.002 | 0.013 |  | -0.031 (0.033) | 0.349 | 0.553 |
| Frontal lobe | 1 | -0.057 (0.045) | 0.203 | 0.464 |  | 0.023 (0.042) | 0.576 | 0.730 |  | 0.038 (0.035) | 0.273 | 0.551 |
|  | 2 | -0.060 (0.045) | 0.180 | 0.432 |  | 0.021 (0.042) | 0.606 | 0.720 |  | 0.038 (0.035) | 0.279 | 0.553 |
| Gray matter | 1 | -0.029 (0.044) | 0.506 | 0.732 |  | 0.030 (0.042) | 0.468 | 0.635 |  | 0.023 (0.035) | 0.515 | 0.652 |
|  | 2 | -0.038 (0.044) | 0.391 | 0.581 |  | 0.026 (0.042) | 0.526 | 0.714 |  | 0.021 (0.035) | 0.553 | 0.657 |
| Hippocampus | 1 | 0.063 (0.045) | 0.166 | 0.464 |  | 0.199 (0.043) | <0.001 | <0.001 |  | 0.152 (0.035) | <0.001 | <0.001 |
|  | 2 | 0.054 (0.045) | 0.239 | 0.432 |  | 0.196 (0.043) | <0.001 | <0.001 |  | 0.150 (0.036) | <0.001 | <0.001 |
| Nucleus accumbens | 1 | 0.025 (0.046) | 0.593 | 0.732 |  | 0.019 (0.043) | 0.663 | 0.741 |  | 0.067 (0.036) | 0.066 | 0.251 |
|  | 2 | 0.012 (0.046) | 0.794 | 0.861 |  | 0.012 (0.043) | 0.783 | 0.826 |  | 0.063 (0.036) | 0.081 | 0.308 |
| Parietal lobe | 1 | -0.052 (0.047) | 0.274 | 0.506 |  | -0.001 (0.045) | 0.989 | 0.989 |  | 0.027 (0.037) | 0.471 | 0.652 |
|  | 2 | -0.054 (0.047) | 0.250 | 0.432 |  | -0.006 (0.045) | 0.899 | 0.899 |  | 0.027 (0.037) | 0.473 | 0.657 |
| Supra tentorial | 1 | 0.002 (0.079) | 0.981 | 0.981 |  | 0.113 (0.075) | 0.132 | 0.279 |  | 0.120 (0.062) | 0.052 | 0.247 |
|  | 2 | 0.028 (0.079) | 0.720 | 0.855 |  | 0.144 (0.076) | 0.058 | 0.138 |  | 0.133 (0.063) | 0.034 | 0.162 |
| Temporal lobe | 1 | -0.043 (0.041) | 0.293 | 0.506 |  | -0.019 (0.039) | 0.631 | 0.741 |  | -0.019 (0.032) | 0.552 | 0.656 |
|  | 2 | -0.047 (0.041) | 0.247 | 0.432 |  | -0.021 (0.039) | 0.595 | 0.720 |  | -0.020 (0.032) | 0.526 | 0.657 |
| Thalamus | 1 | 0.019 (0.056) | 0.729 | 0.815 |  | 0.109 (0.052) | 0.036 | 0.098 |  | 0.004 (0.044) | 0.920 | 0.920 |
|  | 2 | 0.013 (0.056) | 0.816 | 0.861 |  | 0.116 (0.052) | 0.026 | 0.071 |  | 0.001 (0.044) | 0.976 | 0.976 |
| Ventricle | 1 | 0.011 (0.038) | 0.776 | 0.819 |  | -0.099 (0.035) | 0.005 | 0.019 |  | -0.065 (0.030) | 0.029 | 0.184 |
|  | 2 | 0.001 (0.038) | 0.986 | 0.986 |  | -0.112 (0.036) | 0.002 | 0.013 |  | -0.072 (0.030) | 0.018 | 0.114 |
| White matter | 1 | 0.027 (0.052) | 0.599 | 0.732 |  | 0.052 (0.050) | 0.291 | 0.503 |  | 0.046 (0.041) | 0.262 | 0.551 |
|  | 2 | 0.042 (0.052) | 0.422 | 0.581 |  | 0.067 (0.050) | 0.179 | 0.340 |  | 0.052 (0.041) | 0.208 | 0.520 |
| WMH | 1 | 0.103 (0.084) | 0.220 | 0.464 |  | -0.221 (0.079) | 0.005 | 0.019 |  | -0.092 (0.066) | 0.166 | 0.526 |
|  | 2 | 0.104 (0.084) | 0.216 | 0.432 |  | -0.221 (0.080) | 0.006 | 0.023 |  | -0.091 (0.067) | 0.175 | 0.520 |

Values are estimated coefficients (standard error, SE). Model 1 was adjusted for age, sex, years of education, time interval and standardized total intracranial volume; Model 2 was further adjusted for hypertension, diabetes, hyperlipidemia, smoking and alcohol consumption. Abbreviations: FDR, false discovery rate; MMSE, Mini-Mental State Examination; MoCA, Montreal Cognitive Assessment; WMH, white matter hyperintensity.

**Table S3 Association of brain region volume with specific cognitive domain**

| Brain region | Model | Visuospatial function | | |  | Short delay recall | | |  | Long delay recall | | |  | Executive function | | |  | Language | | |
| --- | --- | --- | --- | --- | --- | --- | --- | --- | --- | --- | --- | --- | --- | --- | --- | --- | --- | --- | --- | --- |
|  |  | Beta (SE) | *P* value | *P* value (FDR) |  | Beta (SE) | *P* value | *P* value (FDR) |  | Beta (SE) | *P* value | *P* value (FDR) |  | Beta (SE) | *P* value | *P* value (FDR) |  | Beta (SE) | *P* value | *P* value (FDR) |
| Amygdala | 1 | 0.076 (0.097) | 0.430 | 0.799 |  | -0.036 (0.050) | 0.469 | 0.713 |  | -0.072 (0.052) | 0.165 | 0.314 |  | -0.039 (0.065) | 0.546 | 0.798 |  | 0.045 (0.015) | 0.002 | 0.028 |
|  | 2 | 0.066 (0.098) | 0.503 | 0.816 |  | -0.034 (0.050) | 0.490 | 0.819 |  | -0.073 (0.052) | 0.161 | 0.306 |  | -0.039 (0.065) | 0.550 | 0.804 |  | 0.046 (0.015) | 0.002 | 0.028 |
| Brain Stem | 1 | -0.178 (0.103) | 0.082 | 0.575 |  | 0.017 (0.052) | 0.745 | 0.885 |  | 0.086 (0.055) | 0.117 | 0.300 |  | -0.030 (0.068) | 0.660 | 0.896 |  | -0.003 (0.016) | 0.863 | 0.965 |
|  | 2 | -0.176 (0.104) | 0.090 | 0.592 |  | 0.015 (0.053) | 0.777 | 0.833 |  | 0.089 (0.055) | 0.105 | 0.299 |  | -0.033 (0.068) | 0.629 | 0.854 |  | -0.001 (0.016) | 0.938 | 0.984 |
| Cerebrospinal fluid | 1 | -0.092 (0.092) | 0.316 | 0.799 |  | -0.017 (0.047) | 0.715 | 0.885 |  | -0.072 (0.049) | 0.142 | 0.300 |  | -0.055 (0.061) | 0.367 | 0.757 |  | -0.020 (0.014) | 0.164 | 0.726 |
|  | 2 | -0.123 (0.093) | 0.187 | 0.592 |  | -0.018 (0.047) | 0.707 | 0.833 |  | -0.078 (0.049) | 0.109 | 0.299 |  | -0.063 (0.061) | 0.307 | 0.712 |  | -0.022 (0.014) | 0.128 | 0.547 |
| Frontal lobe | 1 | 0.043 (0.096) | 0.659 | 0.799 |  | -0.078 (0.051) | 0.121 | 0.548 |  | -0.046 (0.053) | 0.385 | 0.610 |  | -0.011 (0.066) | 0.868 | 0.904 |  | 0.008 (0.015) | 0.597 | 0.902 |
|  | 2 | 0.050 (0.097) | 0.607 | 0.816 |  | -0.075 (0.050) | 0.137 | 0.651 |  | -0.045 (0.053) | 0.395 | 0.625 |  | -0.017 (0.066) | 0.802 | 0.902 |  | 0.008 (0.015) | 0.612 | 0.860 |
| Gray matter | 1 | -0.007 (0.096) | 0.945 | 0.945 |  | -0.035 (0.050) | 0.488 | 0.713 |  | -0.001 (0.052) | 0.986 | 0.989 |  | -0.019 (0.065) | 0.770 | 0.904 |  | 0.001 (0.015) | 0.951 | 0.992 |
|  | 2 | 0.0001 (0.097) | 0.996 | 0.996 |  | -0.032 (0.050) | 0.517 | 0.819 |  | -0.003 (0.052) | 0.947 | 0.973 |  | -0.026 (0.065) | 0.694 | 0.879 |  | 0.001 (0.015) | 0.958 | 0.984 |
| Hippocampus | 1 | 0.156 (0.101) | 0.121 | 0.575 |  | -0.010 (0.051) | 0.839 | 0.938 |  | 0.021 (0.053) | 0.695 | 0.932 |  | 0.135 (0.066) | 0.041 | 0.673 |  | 0.020 (0.015) | 0.191 | 0.726 |
|  | 2 | 0.140 (0.102) | 0.170 | 0.592 |  | -0.021 (0.051) | 0.686 | 0.833 |  | 0.003 (0.054) | 0.952 | 0.973 |  | 0.140 (0.067) | 0.035 | 0.659 |  | 0.021 (0.015) | 0.174 | 0.551 |
| Nucleus accumbens | 1 | 0.046 (0.103) | 0.655 | 0.799 |  | -0.043 (0.052) | 0.412 | 0.713 |  | -0.007 (0.055) | 0.895 | 0.989 |  | -0.010 (0.068) | 0.888 | 0.904 |  | 0.0001 (0.016) | 0.992 | 0.992 |
|  | 2 | 0.029 (0.104) | 0.780 | 0.889 |  | -0.046 (0.052) | 0.378 | 0.798 |  | -0.022 (0.055) | 0.687 | 0.870 |  | -0.012 (0.068) | 0.855 | 0.902 |  | 0.0001 (0.016) | 0.984 | 0.984 |
| Parietal lobe | 1 | -0.036 (0.104) | 0.729 | 0.815 |  | -0.062 (0.053) | 0.248 | 0.589 |  | -0.054 (0.056) | 0.330 | 0.570 |  | 0.016 (0.069) | 0.817 | 0.904 |  | -0.006 (0.016) | 0.686 | 0.931 |
|  | 2 | -0.026 (0.104) | 0.804 | 0.889 |  | -0.057 (0.053) | 0.290 | 0.689 |  | -0.055 (0.056) | 0.326 | 0.563 |  | 0.013 (0.070) | 0.855 | 0.902 |  | -0.008 (0.016) | 0.634 | 0.860 |
| Supra tentorial | 1 | 0.198 (0.173) | 0.252 | 0.799 |  | -0.104 (0.089) | 0.244 | 0.589 |  | -0.070 (0.093) | 0.449 | 0.656 |  | 0.092 (0.116) | 0.427 | 0.757 |  | 0.020 (0.027) | 0.444 | 0.902 |
|  | 2 | 0.256 (0.177) | 0.148 | 0.592 |  | -0.108 (0.090) | 0.226 | 0.689 |  | -0.055 (0.094) | 0.555 | 0.811 |  | 0.111 (0.117) | 0.345 | 0.712 |  | 0.025 (0.027) | 0.355 | 0.802 |
| Temporal lobe | 1 | -0.049 (0.089) | 0.580 | 0.799 |  | -0.018 (0.046) | 0.700 | 0.885 |  | -0.001 (0.048) | 0.989 | 0.989 |  | -0.058 (0.060) | 0.329 | 0.757 |  | -0.003 (0.014) | 0.820 | 0.965 |
|  | 2 | -0.041 (0.090) | 0.644 | 0.816 |  | -0.017 (0.046) | 0.716 | 0.833 |  | -0.002 (0.048) | 0.973 | 0.973 |  | -0.063 (0.060) | 0.297 | 0.712 |  | -0.003 (0.014) | 0.822 | 0.984 |
| Thalamus | 1 | -0.021 (0.122) | 0.866 | 0.914 |  | -0.008 (0.063) | 0.900 | 0.950 |  | -0.005 (0.066) | 0.934 | 0.989 |  | 0.111 (0.082) | 0.177 | 0.673 |  | -0.005 (0.019) | 0.799 | 0.965 |
|  | 2 | -0.024 (0.123) | 0.842 | 0.889 |  | -0.017 (0.063) | 0.789 | 0.833 |  | -0.004 (0.066) | 0.948 | 0.973 |  | 0.106 (0.082) | 0.200 | 0.659 |  | -0.003 (0.019) | 0.862 | 0.984 |
| Ventricle | 1 | -0.081 (0.087) | 0.354 | 0.799 |  | -0.062 (0.043) | 0.149 | 0.548 |  | -0.133 (0.045) | 0.003 | 0.057 |  | -0.061 (0.056) | 0.273 | 0.757 |  | -0.015 (0.013) | 0.237 | 0.750 |
|  | 2 | -0.103 (0.089) | 0.248 | 0.673 |  | -0.058 (0.043) | 0.177 | 0.673 |  | -0.142 (0.045) | 0.002 | 0.038 |  | -0.071 (0.056) | 0.208 | 0.659 |  | -0.019 (0.013) | 0.144 | 0.547 |
| White matter | 1 | 0.087 (0.113) | 0.440 | 0.799 |  | 0.002 (0.059) | 0.979 | 0.979 |  | 0.021 (0.061) | 0.736 | 0.932 |  | 0.056 (0.076) | 0.458 | 0.757 |  | 0.012 (0.017) | 0.479 | 0.902 |
|  | 2 | 0.106 (0.114) | 0.354 | 0.816 |  | -0.001 (0.059) | 0.981 | 0.981 |  | 0.029 (0.061) | 0.638 | 0.866 |  | 0.067 (0.076) | 0.375 | 0.712 |  | 0.015 (0.018) | 0.400 | 0.802 |
| WMH | 1 | -0.087 (0.186) | 0.640 | 0.799 |  | -0.161 (0.096) | 0.094 | 0.548 |  | -0.231 (0.100) | 0.021 | 0.133 |  | -0.180 (0.126) | 0.153 | 0.673 |  | -0.021 (0.029) | 0.474 | 0.902 |
|  | 2 | -0.128 (0.189) | 0.497 | 0.816 |  | -0.146 (0.097) | 0.131 | 0.651 |  | -0.236 (0.101) | 0.020 | 0.190 |  | -0.185 (0.127) | 0.147 | 0.659 |  | -0.024 (0.029) | 0.419 | 0.802 |

Values are estimated coefficients (standard error, SE). Model 1 was adjusted for age, sex, years of education, time interval and standardized total intracranial volume; Model 2 was further adjusted for hypertension, diabetes, hyperlipidemia, smoking and alcohol consumption. Abbreviations: FDR, false discovery rate; WMH, white matter hyperintensity.

**Table S4 Summary of measurements**

| **Exposure category** | **Measurements (variables)** |
| --- | --- |
| Demographics | Questionnaire (sex; age; education) |
| Pre-existing comorbidity | Questionnaire and interview (hypertension; diabetes; hyperlipidemia) |
| Lifestyle | Questionnaire and interview (smoking history; alcohol drinking history) |
| Neuropsychological assessment | MMSE and MoCA (global cognitive function); Chinese version of Auditory Verbal Learning Test (short delay recall and long delay recall); Modified Fuld Object Memory Evaluation (short delay recall and long delay recall); Conflicting Instructions Task (executive function); Clock Drawing Test (visuospatial function); Animal Fluency Test (language) |
| Olfactory function assessment | 12-item Sniffin’s Sticks screening test (olfactory identification) |

Abbreviations: MMSE, Mini-Mental State Examination; MoCA, Montreal Cognitive Assessment.

**Table S5 Stratified association of olfactory identification with cognitive function**

|  | MMSE | | | MoCA | | Language | | |
| --- | --- | --- | --- | --- | --- | --- | --- | --- |
|  | Beta (SE) | *P* value | Beta (SE) | | *P* value | | Beta (SE) | *P* value |
| Olfactory identification | 0.068 (0.017) | <0.001 | 0.098 (0.014) | | <0.001 | | 0.332 (0.070) | <0.001 |
| **Amygdala volume in the top 50%** |  |  |  | |  | |  |  |
| Olfactory identification | 0.078 (0.025) | 0.002 | 0.119 (0.020) | | <0.001 | | 0.304 (0.103) | 0.003 |
| **Amygdala volume in the last 50%** |  |  |  | |  | |  |  |
| Olfactory identification | 0.063 (0.023) | 0.006 | 0.089 (0.019) | | <0.001 | | 0.401 (0.093) | <0.001 |

Values are estimated coefficients (standard error). All models were adjusted for age, sex, years of education, hypertension, diabetes, hyperlipidemia, smoking and alcohol consumption. Abbreviations: MMSE, Mini-Mental State Examination; MoCA, Montreal Cognitive Assessment.

**Figure Legends**

**Figure S1 Flowchart of subject selection**
